# Assessment of a statistical AIF extraction method for dynamic PET studies with ^15^O water and ^18^F fluorodeoxyglucose in locally advanced breast cancer patients

**DOI:** 10.1117/1.JMI.5.1.011010

**Published:** 2017-11-23

**Authors:** Finbarr O’Sullivan, Janet N. O’Sullivan, Jian Huang, Robert Doot, Mark Muzi, Erin Schubert, Lanell Peterson, Lisa K. Dunnwald, David M. Mankoff

**Affiliations:** a University College Cork, School of Mathematical Sciences, Cork, Ireland; b University of Pennsylvania, Department of Radiology, Philadelphia, Pennsylvania, United States; c University of Washington, Department of Radiology, Seattle, Washington, United States; d University of Iowa, Department of Radiology, Iowa City, Iowa, United States

**Keywords:** AIF extraction, breast cancer, blood flow-metabolism mismatch, physiologically based pharmacokinetic model, kinetic analysis, positron emission tomography

## Abstract

Blood flow-metabolism mismatch from dynamic positron emission tomography (PET) studies with O15-labeled water ($H_{2}O$) and F18-labeled fluorodeoxyglucose (FDG) has been shown to be a promising diagnostic for locally advanced breast cancer (LABCa) patients. The mismatch measurement involves kinetic analysis with the arterial blood time course (AIF) as an input function. We evaluate the use of a statistical method for AIF extraction (SAIF) in these studies. Fifty three LABCa patients had dynamic PET studies with $H_{2}O$ and FDG. For each PET study, two AIFs were recovered, an SAIF extraction and also a manual extraction based on a region of interest placed over the left ventricle (LV-ROI). Blood flow-metabolism mismatch was obtained with each AIF, and kinetic and prognostic reliability comparisons were made. Strong correlations were found between kinetic assessments produced by both AIFs. SAIF AIFs retained the full prognostic value, for pathologic response and overall survival, of LV-ROI AIFs.

## 1 Introduction

The role for positron emission tomography (PET) imaging in oncology is expanding with a range of clinical protocols under consideration for many specific cancers. Blood flow-metabolism mismatch from dynamic PET studies, e.g., with O15-labeled water ($H_{2}O$) and F18-labeled fluorodeoxyglucose (FDG), has a history in the cardiac applications^1^ but has also shown potential as a diagnostic biomarker for the management of patients with locally advanced breast cancer (LABCa).^2^ Realization of this measurement involves kinetic analysis of dynamically acquired $H_{2}O$ and FDG data with the time courses of the PET tracers in arterial blood (AIF) as input functions. While direct recovery of an AIF by catheterized arterial sampling is a gold standard, it is not routinely feasible in most clinical settings. As a result, image-based extraction of the AIF is required. In breast cancer studies, the imaging field of view encompasses the heart, so it is possible to recover an estimate of the AIF by careful placement of a region of interest (ROI) over an appropriate part of the left ventricle (LV). We refer to this as the LV-ROI method. But extraction of the AIF signal from ROIs drawn over the LV can be challenging and hard to reproduce, even for a highly experienced analyst. The finite resolution of PET and the motion of the heart and lung during scanning create spillover artifacts in time-course data derived from LV regions. With FDG, late tracer uptake in myocardial tissue surrounding the LV is an obvious source^3^ of spillover but there may be others as well. For example, at earlier times, the spillover from nonarterial blood pools in the right ventricle (RV) and the lung can play a role. Simplified and reproducible determination of the AIF from the image data would facilitate more widespread consideration of blood flow-metabolism mismatch for clinical management of LABCa. The present work is focused on that goal.

Two main approaches have been proposed as alternatives to catheterized arterial sampling for determination of the AIF in a dynamic PET study. The fully image-based approach focuses on arterial signals associated with blood pools largely using manually placed ROIs over corresponding blood pool structures in the imaging field of view, with possible corrections to account for the contaminating effects of spillover of tracer uptake into these areas from surrounding tissue.^4^^,^^5^ The population-template approach uses a standard reference or template AIF, typically obtained by averaging a population of directly sampled historical AIFs. The population template is scaled to the study at hand by a direct blood sample, or scaling is derived from an image measurement for a blood pool in the field of view of the scanner.^6^^,^^7^ Our group has developed a hybrid statistical AIF extraction (SAIF) method that attempts to combine aspects of the fully image-based and the population-template approaches. SAIF makes use of a physiologically based pharamocological model of tracer circulation within the body. It uses a Bayesian formulation to take account of prior AIF information.^8^ The method has the advantage of formulating the recovery of the AIF in such a way that it is possible to make use of information about the AIF from nonarterial as well as arterial sources. In particular, for breast cancer studies, when the heart and lungs are in the field of view, information from the lung, RV, and more general venous blood signals can positively contribute to the AIF estimation—as opposed to being regarded as undesirable sources of contamination. The approach has shown promise in the context of cerebral PET studies with $H_{2}O$ and FDG^8^ where no suitably large blood pool structure is in the imaging field of view. This paper presents an assessment of SAIF in the context of blood flow-metabolism mismatch evaluation for LABCa studies, where classical image-based AIF recovery from manually placed ROIs in the LV by a skilled operator familiar with the anatomy can be technically challenging and difficult to reproduce.

## 2 Review of the SAIF Extraction Technique

### 2.1 Model Representation of the AIF

**Fig. 1:**
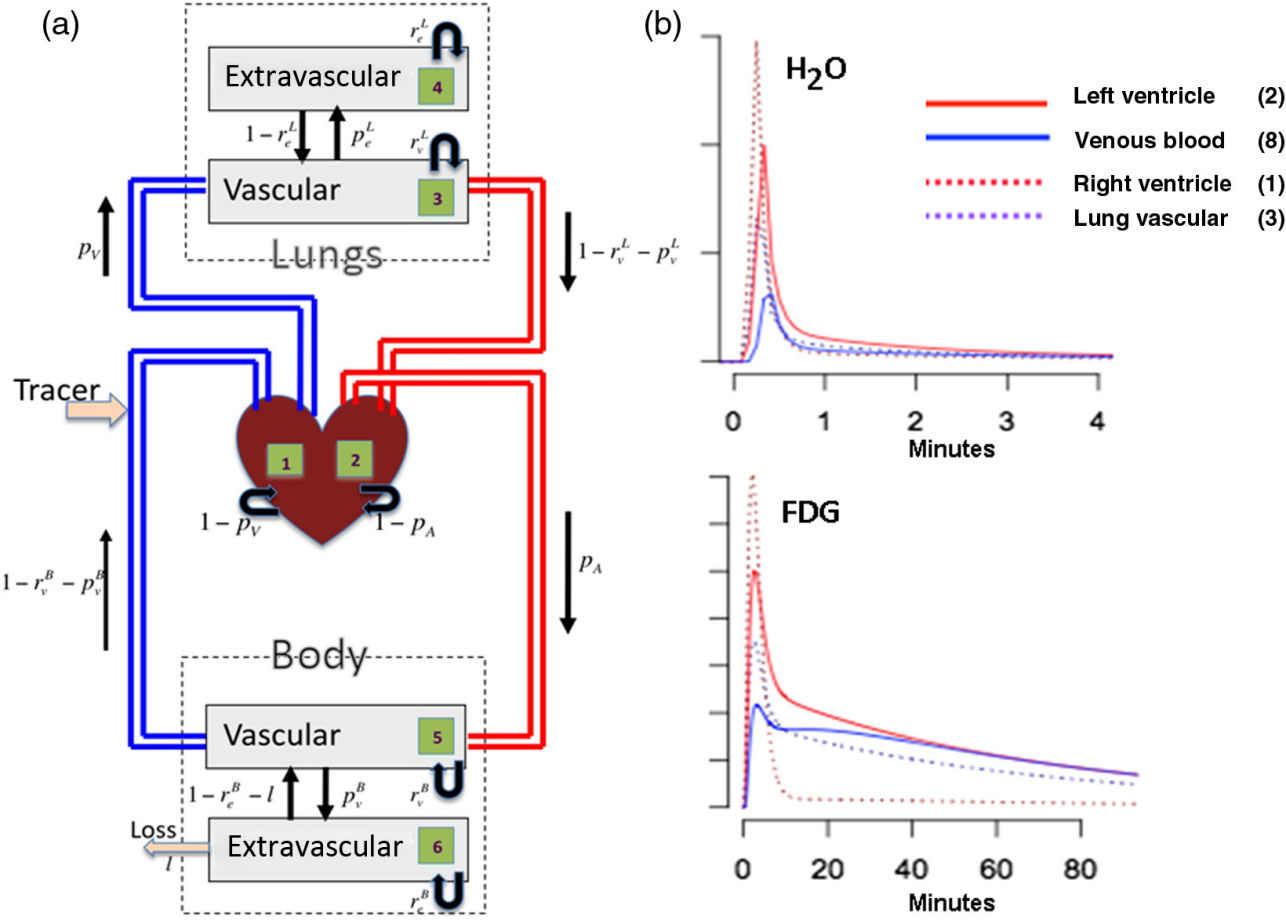
(a) Whole-body circulation model for tracer atom dynamics within the body. The model has eight states detailed at the beginning of Sec. 2. States 1 to 6 are indicated with numbers in schematic, the other two states are loss output (lower-left, state 7) and venous blood (blue, state 8). The parameters in the model, θ in text, determine the various transition probabilities associated with the black arrows. (b) Illustrative time-activity curve profiles for regions—associated states and numbers indicated—are displayed for $H_{2}O$ (O15-labeled $H_{2}O$) and FDG (F18-labeled FDG). The injection profile durations are 5 s ($H_{2}O$) and 2 min (FDG). In the plots, the RV profile is normalized to have a maximum of unity with other profiles scaled relative to it.

A physiologically based pharmacokinetic Markov chain model^9^^,^^10^ is used to describe tracer atom movement with the systematic circulation (see Fig. 1). There are eight different states, each representing different parts of the body: (1) RV, (2) LV (arterial blood), (3) lung vasculature, (4) lung extravascular space, (5) body vasculature, (6) body extravascular space, (7) loss blood, and (8) venous blood. The random movement of a tracer atom between model states on each time step (heart beat) is governed by a transition matrix. The Markov chain transition matrix is

$$P=[\begin{array}{cccccccc} 1-p_{c} & 0 & p_{c} & 0 & 0 & 0 & 0 & 0\\ 0 & 1-p_{c} & 0 & 0 & p_{c} & 0 & 0 & 0\\ 0 & 1-r_{v}^{L}-p_{e}^{L} & r_{v}^{L} & p_{e}^{L} & 0 & 0 & 0 & 0\\ 0 & 0 & 1-r_{e}^{L} & r_{e}^{L} & 0 & 0 & 0 & 0\\ 0 & 0 & 0 & 0 & r_{v}^{B} & p_{e}^{B} & 0 & 1-r_{v}^{B}-p_{e}^{B}\\ 0 & 0 & 0 & 0 & 1-r_{e}^{B}-l & r_{e}^{B} & l & 0\\ 0 & 0 & 0 & 0 & 0 & 0 & 1 & 0\\ p_{c} & 0 & 0 & 0 & 0 & 0 & 0 & 1-p_{c} \end{array}],$$ (1)

where the rows and columns are ordered by states (1 to 8). Note $P_{ij}$, here, represents the probability that a tracer atom moves to state i at the next time step, given that it is currently in state j. The requirement that the transitions must be probabilities with a unitary row sum imposes a number of constraints. A set of the transformations is used in parameterization of the transition matrix

$$o_{r}=\frac{r_{v}^{L}}{r_{v}^{B}};\;r=r_{v}^{L}+r_{v}^{B}o_{d}=\frac{1-r_{e}^{L}}{1-r_{e}^{B}-\ell };\;d=1-r_{e}^{L}+1-r_{e}^{B}-\ell o_{L}=\frac{p_{e}^{L}}{1-r_{v}^{L}-p_{e}^{L}};\;o_{B}=\frac{p_{e}^{B}}{1-r_{v}^{B}-p_{e}^{B}}o_{s}=\frac{l}{r_{e}^{B}};\;e^{\prime }=\frac{o_{L}}{o_{B}};\;e=o_{L}+o_{B}.$$ (2)

It is also required that $r=r_{v}^{L}+r_{v}^{B}$ and $d=1-r_{e}^{L}+1-r_{e}^{B}-\ell$ be nonnegative. The input to the Markov model is represented by the convolution of a specified injection profile (typically a square wave of known duration) and a gamma density with shape and scale (α,β)—the choice of the gamma density, here, is based on empirical performance. In addition, there is a nonnegative scale (A) and a delay parameter (Δ) that adapts to the timing of the injection relative to the measured PET data. Using a combination of logarithmic and logistic transforms, the model is parameterized by a vector $\theta =(\theta_{\Delta },\theta_{A},\theta_{\alpha },\theta_{\beta },\theta_{c},\theta_{r},\theta_{or},\theta_{d},\theta_{od},\theta_{e},\theta_{e^{\prime }},\theta_{s})$. The elements of θ are mapped to the unknowns according to

$$\Delta =\theta_{\Delta };\;A=e^{\theta_{A}};\;\alpha =e^{\theta_{\alpha }};\;\beta =e^{\theta_{\beta }};\;p_{c}=\frac{e^{\theta_{c}}}{1+e^{\theta_{c}}}$$

and, with

$$r=\frac{e^{\theta_{r}}}{1+e^{\theta_{r}}};\;o_{r}=e^{\theta_{or}};\;d=\frac{e^{\theta_{d}}}{1+e^{\theta_{d}}};\;o_{d}=e^{\theta_{od}}\;e=e^{\theta_{e}};\;e^{\prime }=e^{\theta_{e^{\prime }}};\;o_{s}=e^{\theta_{s}},$$

further transition matrix elements are given by

$$r_{v}^{L}=r\frac{o_{r}}{1+o_{r}};\;r_{v}^{B}=r\frac{1}{1+o_{r}}r_{e}^{L}=1-d\frac{o_{d}}{1+o_{d}};\;r_{e}^{B}=(1-d\frac{1}{1+o_{d}})\frac{1}{1+o_{s}}\;p_{e}^{L}=(1-r_{v}^{L})\frac{o_{L}}{1+o_{L}};\;p_{e}^{B}=(1-r\frac{1}{1+o_{r}})\frac{o_{B}}{1+o_{B}}\;\ell =(1-d\frac{1}{1+o_{d}})\frac{o_{s}}{1+o_{s}},$$ (3)

where $o_{L}=e\frac{e^{\prime }}{1+e^{\prime }}$ and $o_{B}=e\frac{1}{1+e^{\prime }}$. The net result of the above is that with a specified value for the parameter vector θ and a given injection profile, the time course for the amount of tracer in each state, including the arterial blood time course or AIF, can be evaluated.^8^

### 2.2 Estimation Method

Based on the above, the estimation of the AIF is replaced by a problem of estimating θ. Information from the available image data as well as *a-priori* past experience with the tracer is both used in a combined Bayesian-like estimation strategy. It is important to appreciate that while the circulation model references the whole body, the extraction procedure only requires data from the field of view of the scan—not the whole body. The imaging data are reduced by segmentation/clustering to obtain a statistically representative collection of time courses. The exact number of time courses is selected adaptively based on the characteristics of the image data. Details of the segmentation techniques are given in Refs. 11 and 12—a combination of split-and-merge segmentation and k-means clustering is used. Note the present AIF extraction, including the segmentation analysis, is applied separately to the $H_{2}O$ and FDG studies. A combined approach could potentially enhance the performance. Each of the segmentation-derived time courses, denoted as $z_{j}(t)$ with j indicating the segment number and t the time frame, is modeled as a positive linear combination of the eight profiles generated by the PKPB model as well as a cumulative AIF profile, i.e., a Patlak term, representing tissue retention. For a given θ value, the model-derived prediction of the segment time course is denoted as z^j(t|θ). Note that z^j(t|θ) is the prediction of $z_{j}(t)$ based on the best positive linear combination of the θ-based PKPB model profiles. By fitting the PKPB model to directly sample arterially time courses in historical studies, i.e., prior experience, an *a-priori* characterization of the population behavior of the parameter vector θ can be created. Using p to signify population, we compute the population mean ($\mu_{p}$) and covariance matrix ($\Sigma_{p}$) of $\theta^{*}$. Here, $\theta^{*}$ is defined as the part of θ with scale ($\theta_{A}$) and delay ($\theta_{\Delta }$) elements removed. Our estimation of θ is based on an objective function that combines the information from the imaging data at hand and the historical experience relevant to the tracer. The optimal parameter choice, θ^, is chosen to minimize the objective function

ℓλ(θ)=∑j=1J∑t=1Twj(t)[zj(t)−z^j(t|θ)]2+λ(θ*−μp)′Σp−1(θ*−μp). (4)

This can be viewed as a Bayesian technique.^6^ λ is a positive scale factor, which is adjusted to ensure that the estimate remains consistent with *a-priori* experience. Specifically, the value of λ is adjusted to ensure that the Mahalanobis distance of the nonscale and delay parts of the estimate (θ^*) from the *a-priori* mean ($\mu_{p}$), i.e., (θ^*−μp)′Σp−1(θ^*−μp), does not exceed the 90th percentile of corresponding distance values observed in historical data. The weights [$w_{j}(t)$] used reflect the reliability of the PET data—this is based on the approximate Poisson nature of the measurement.^13^ We also adjust the weighting to place more emphasis on the early part of time course (within the first 4 min for $H_{2}O$ and 15 min for FDG) where vascular signals are more dominant. The entire approach is implemented with substantial reliance on open-source software, specifically AMIDE^14^ and R.^15^

### 2.3 Scaling

The above estimation process does not reliably identify the absolute scale of the AIF. A separate process is used to scale. Since arterial and venous blood profiles ultimately equilibrate,^8^ it is possible to scale our AIF curve using either arterial or venous sampled data. As long as the timing of these measurements is known relative to the injection time of the tracer, there is no need to restrict to later sampling time points. In particular, the sampling times do not have to be taken at a time when the arterial and venous activities can be considered to have equilibrated. This flexibility allows the blood sampling to occur when the activity levels are high and the relative error in the blood measurements is lower. In the context of blood flow-metabolism mismatch studies, a scaling based on data recovered from measurements made in LV-ROIs or possibly even the descending aorta is certainly feasible, including at early- or late-time points where spillover from adjacent structure does not pose a challenge (early) or arterial–venous equilibrium minimizes the impact of spillover between arterial and venous structures. In the LABCa studies reported below, AIFs were scaled using late-time blood time-course data recovered from the LV-ROI, and this is to ensure better agreement with the LV-ROI-extracted AIFs.

## 3 Experimental Evaluation

### 3.1 Clinical Study and Data

A dataset from a study of PET imaging in LABCa patients is used. The study is fully described in a number of reports, see, for example, Dunnwald et al.^2^ and Mankoff et al.^16^ Briefly, this was an institutional review board-approved prospective study at the University of Washington Medical Center. Patients with histologically confirmed breast carcinoma and scheduled for neoadjuvant chemotherapy prior to surgical resection were eligible. Selected patients were imaged at baseline before chemotherapy and also at mid- and posttherapy. Patients underwent definitive breast and axillary surgery posttherapy where any remaining tumor was resected. Our analysis focuses on the baseline data from the set of 53 patients. As previously reported, the patient data include standard clinical diagnostics, including the number of positive axillary nodes and the estrogen receptor (ER) and progesterone receptor (PR) status.^16^ Outcome data for treatment for each patient were recorded as the presence or absence of residual tumor at posttherapy surgery [no pathologic complete response (pCR) versus pCR], disease-free survival, and overall survival (OS). Survival and disease progression were assessed^16^ from the time of diagnosis. The date of the last follow-up is May 4, 2009, and patients who had not died or progressed at that point were considered censored in the analysis.

### 3.2 PET Imaging and Data Processing

Details of PET radiotracer production and dynamic tumor imaging protocols are given in the earlier reports.^2^^,^^16^ For $H_{2}O$, 725 to 1902 MBq of [O15]-labeled $H_{2}O$ in a 1- to 4-mL volume was injected as a bolus; with FDG, 218 to 396 MBq of [F18]-labeled FDG in 7- to 10-mL volume was injected over 2 min with a constant infusion pump. The $H_{2}O$ and FDG imaging studies were conducted in the same session on a 35-plane GE-Advance scanner, starting with the $H_{2}O$ study. The axial extent of the scanner field of view is roughly 20 cm. Dynamic $H_{2}O$ imaging was carried out over 8.75 min according to the following acquisition sequence (number of frames and their durations given): 1 (1 min) preinjection, 15 (2 s), 15 (5 s), 12 (10 s), 8 (15 s), and 6 (20 s). The FDG injection and dynamic imaging followed $H_{2}O$. The FDG acquisitions were conducted over 61 min according to the sequence: 1 (1 min) preinjection, 4 (20 s), 4 (40 s), 4 (1 min), 4 (3 min), and 8 (5 min). All data were acquired in two-dimensional mode using a filtered-backprojection reconstruction algorithm. Circular ROIs of ∼1.5-cm diameter were drawn over tumor and contra-lateral normal breast, a similarly sized ROI was placed over a portion of the LV. These ROIs provided tissue and blood time-course (LV-ROI) data for analysis. The SAIF extraction method, reviewed above, was applied to the data to produce a second AIF. AIFs were scaled using late-time LV blood time-course data. A voxel-level analysis of the dynamic data was also applied^17^ to obtain a mapping of the local blood volume for $H_{2}O$ and FDG. Tissue time-course data were analyzed using the standard 1- and 2-compartmental models of Kety for $H_{2}O$^18^ and Huang–Sokoloff for FDG.^19^ All numerical computations, including segmentation, SAIF extraction, and compartmental model fitting by nonlinear least squares were carried out using the open-source R Statistical Computing Software.^15^ The AIFs produced by the LV-ROI and SAIF were used as inputs for the compartmental analysis of tissue time-course data. Estimates of blood flow ($K^{H}$, mL/min/g) and the tissue FDG-based glucose metabolic rate (${\mathrm{MR}}_{\mathrm{glc}}^{F}$, mg/min/100  g of tissue) were then constructed. In addition, the blood flow-metabolism mismatch ratio $\frac{{\mathrm{MR}}_{\mathrm{glc}}^{F}}{K^{H}}$, measuring the glucose utilization rate per unit blood flow, was also created.

### 3.3 Statistical Analysis

Pair-wise plots and correlations between LV-ROI- and SAIF-derived kinetic variables, including the blood flow-metabolism mismatch, were evaluated. The impact of the alternative AIFs on the prediction of patient outcomes, pathologic response, and OS from Dunnwald et al.^2^ was also considered. For survival, a multivariate Cox regression analysis, incorporating standard clinical diagnostics and either LV-ROI- or SAIF-derived blood flow-metabolism mismatch, were compared. Formal comparisons were based on the performance of the prognostic model, likelihood, and concordance values were considered. The statistical Bootstrap methodology^20^ was used to evaluate differences between model performance criteria, appropriately adjusted for bias. Standard errors for differences between LV-ROI and SAIF prognostic models are also reported. A similar approach was used for pathologic response (CR). A multivariate logistic regression incorporating standard clinical diagnostics and either LV-ROI- or SAIF-derived blood flow-metabolism mismatch was compared. The comparisons were based on the likelihood performance (deviance) of the logistic model. All analyses were conduced in the R statistical package.^15^

## 4 Results

### 4.1 Illustrative AIF Reconstructions

**Fig. 2:**
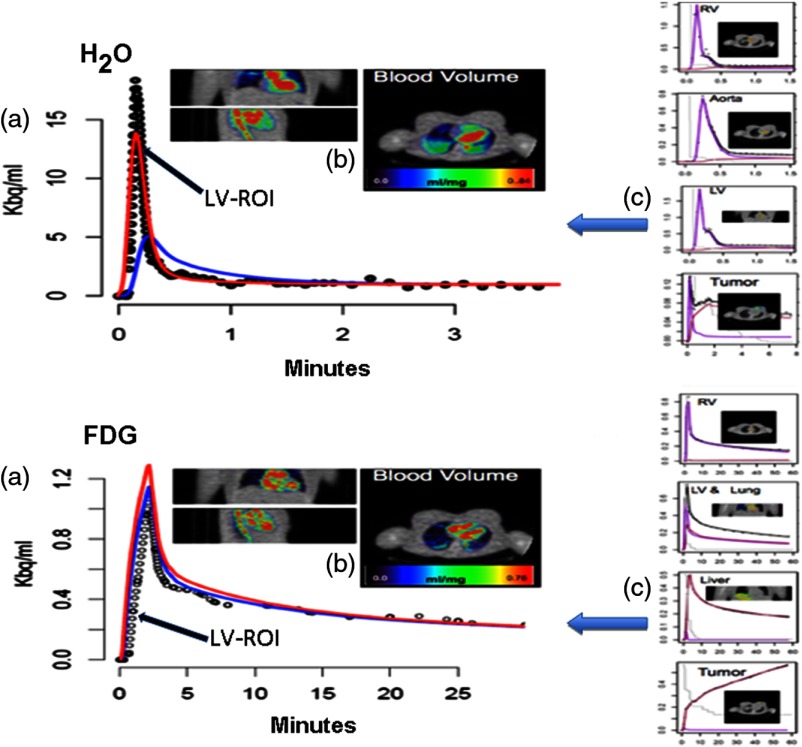
Sample SAIF reconstructions for $H_{2}O$ and FDG studies in a breast cancer patient: (a) estimated SAIF (red) and venous (blue) times-courses, sampled LV data (points); (b) local blood volume maps, produced by residue analysis,^17^ are overlaid on the PET attenuation scan; and (c) segment time-course data (points) with vascular (purple) and extravascular (maroon) components indicated.

Sample SAIF reconstructions for an $H_{2}O$ and FDG study are shown in Fig. 2. Included in this are some of the image-derived time courses produced by the automated data-segmentation procedure during the extraction process. The selected time courses shown originate in regions where arterial (LV), prearterial (RV and lung), and postarterial venous signals dominate. The ability of the SAIF methodology to harvest information from these diverse sources is a key feature of the approach. Comparisons between the LV-ROI and SAIF are shown in Fig. 2. The recovered venous time-course information, a by product of the SAIF technique, is also shown. For $H_{2}O$, the SAIF is not as peaked as the LV-ROI data. This may be due to early spillover from the RV, which receives the initial bolus signal at the heart. With FDG, the tracer injection was over a 2-min period. We expect less impact from the RV on the LV data. The FDG SAIF has a higher peak than the LV-ROI-based AIF. The SAIF is scaled by the tail of the LV measurement. With FDG, a case could be made to scale by the earlier LV data. Such an approach could bring the SAIF into better alignment with the initial phase of the LV-ROI AIF while also reducing its tail height.

### 4.2 Kinetic Analysis

**Fig. 3:**
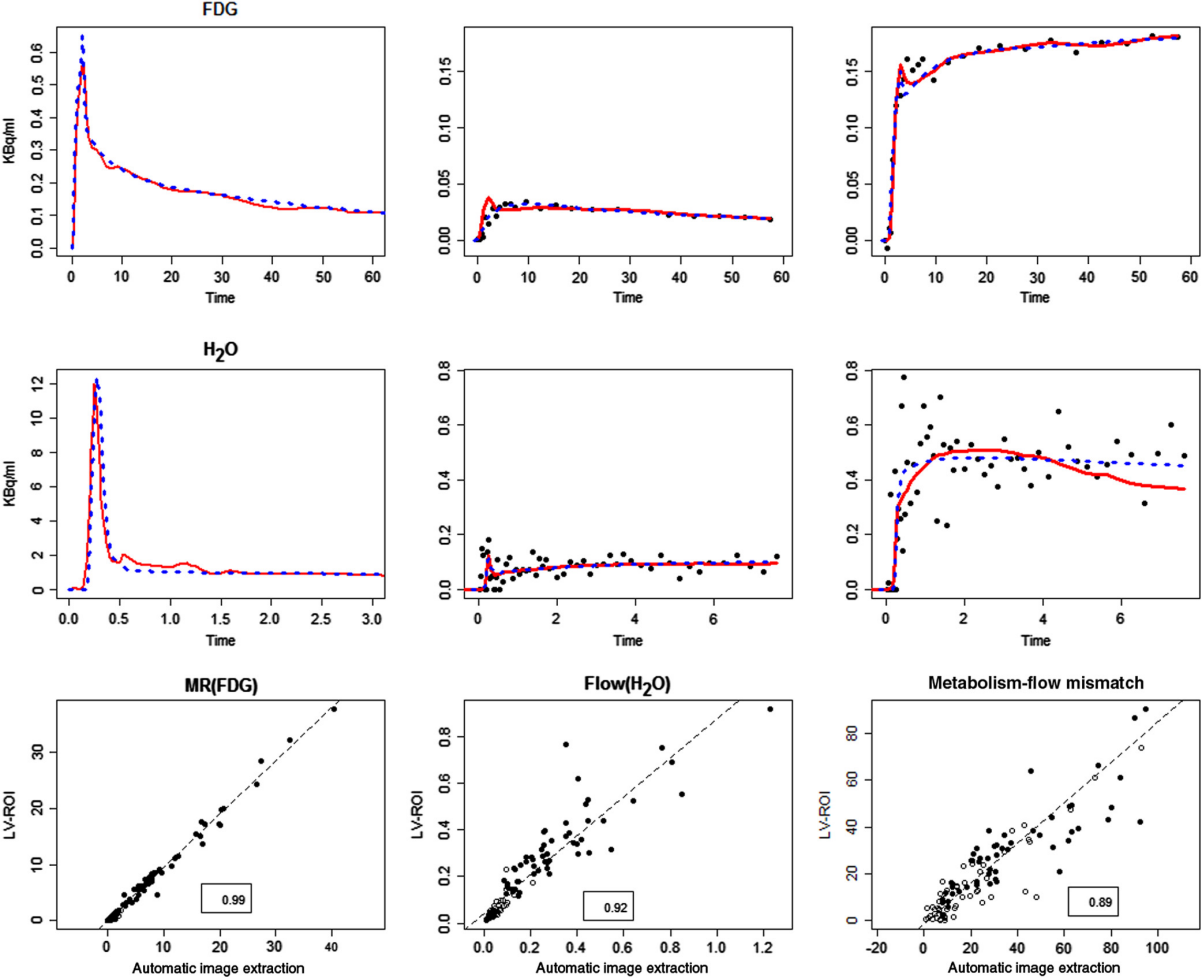
Comparison of LV-ROI and SAIF AIFs in terms of kinetics analysis for tissue data. Top two rows show ROI data versus time (in minutes) for normal (middle) and tumor (right) regions in one patient. AIFs and model fits of ROI data with LV-ROI (solid-red) and SAIF AIFs (dashed-blue) are shown. Kinetic parameter comparisons for the glucose metabolic rate as determined by FDG, MR(FDG) in mg/min per 100 units, blood flow, flow ($H_{2}O$) in mL/min/g units, and the associated blood flow-metabolism mismatch ratio in all 106 ROIs examined are shown in the bottom row (SAIF values on the x-axis)—solid/open dots represent tumor/normal ROIs. Linear correlation coefficients are indicated in boxes.

Time-course data and corresponding kinetic model analysis obtained using the LV-ROI and SAIF AIFs are shown in Fig. 3. The data are from the same study as shown in Fig. 2. In the case of FDG, the LV-ROI and SAIF AIFs deviate by a maximum of 10%; with $H_{2}O$, the maximum deviation is 25%. Figure 3 shows differences between the fits of kinetic models based on the LV-ROI and SAIF inputs. This is most apparent for the $H_{2}O$ data, perhaps to be expected given the variability of the time-course data. Figure 3 also presents comparisons between the $H_{2}O$-derived blood flow, the FDG-derived glucose metabolic rate, and the blood flow-metabolism mismatch. High correlations are observed for all three parameters. Not surprisingly, the strongest relation (r=0.99) is found for glucose metabolic rate. The greater divergence is observed for the $H_{2}O$-derived blood flow (r=0.92). Again, in light of variation in the time-course data for $H_{2}O$, the increased divergence is not surprising. The observed correlation for the blood flow-metabolism mismatch is the weakest (r=0.89), which is still highly satisfactory. Further comparisons between kinetic parameters, including computed blood and tissue volumes of distributions, also show strong association between values produced using LV-ROI and SAIF as input functions.

### 4.3 Prognostic Comparisons

Table 1 reports results of multivariate Cox regression models for OS using established clinical diagnostics and the PET-derived blood flow-metabolism mismatch values produced using the LV-ROI and SAIF AIFs. The established clinical variables are the PR and ER receptor status (negativity) of the tumor (PR and ER) and the number of nodes involved (${\text{Nodes}}^{+}$). The differences between the Cox model regression coefficients obtained with the different AIFs are <20% of the associated estimated standard errors—statistically insignificant. The overall fit of the Cox model in terms of log-likelihood and concordance is also shown in Table 1. The Bootstrap estimates the difference in the Cox model fit are 0.15(±1.90) and 0.007(±0.025) for the log-likelihood and concordance, respectively. These differences are in the order of 1% and nonsignificant in formal statistical terms—p-values are shown in the table. Corresponding results for the analysis of pathologic response using the multivariate logistic regression model are given in Table 2. Again, the differences between the logistic regression model coefficients obtained with the different AIFs are <40% of the associated estimated standard errors—statistically insignificant. The difference in the deviance (negative logarithm of likelihood) of the models is −3.63(±4.18), showing 10% improvement with the SAIF variable, but as with survival analysis, the difference in deviances is statistically insignificant, p-value is 0.34.

**Table 1:** Multivariate Cox regression analysis of time to death. Based on 53 subjects, 10 of whom were reported dead at the last follow-up. Regression model: $\log(\text{Risk of death})=\mathrm{PR}+\sqrt{{\text{Nodes}}^{+}}+\mathrm{ER}+\text{Mismatch}$.

Factor	LV-ROI			SAIF		
	Coef	SE	p-value	Coef	SE	p-value
PR	2.43	1.26	0.09	2.26	1.27	0.08
${\text{Nodes}}^{+}$	0.86	0.27	0.001	0.91	0.28	0.001
ER	−1.14	1.17	0.33	−1.07	1.16	0.35
Mismatch	0.44	0.17	0.01	0.37	0.17	0.03
Model fits	LV-ROI	SAIF	Difference*	SE*	p-value*	
Likelihood	16.55	16.05	0.145	1.90	0.469	
Concordance	0.769	0.776	0.007	0.025	0.388	

* Values based on Bootstrap resampling.^20^

**Table 2:** Multivariate logistic regression analysis of response. Based on 53 subjects, 11 had pathologic responses to chemotherapy. Regression model: $\log(\text{Risk of death})=\mathrm{PR}+\sqrt{{\text{Nodes}}^{+}}+\mathrm{ER}+\text{Mismatch}$.

Factor	LV-ROI			SAIF		
	Coef	SE	p-value	Coef	SE	p-value
PR	−2.12	1.46	0.09	−2.82	1.79	0.11
${\text{Nodes}}^{+}$	0.18	0.33	0.59	0.24	0.34	0.48
ER	−0.35	1.40	0.80	−0.15	1.61	0.92
Mismatch	0.098	0.041	0.02	0.102	0.044	0.02
Model fit	LV-ROI	SAIF	Difference*	SE*	p-value*	
Deviance	39.49	34.75	−3.64	4.17	0.38	

* Values based on Bootstrap resampling.^20^

**Fig. 4:**
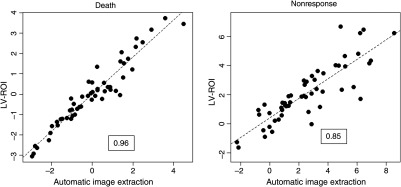
Prognostic comparison. Model-defined risk computed from coefficients in Tables 1 (left) and Table 2 (right) resulting from LV-ROI and SAIF (automatic image extraction) generated blood flow-metabolism mismatch values. Correlation coefficients among risk values are shown in boxes.

**Fig. 5:**
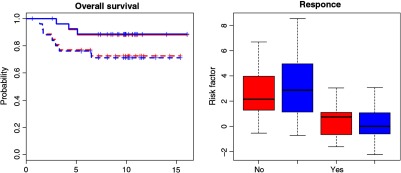
Prognostic comparison: (a) Kaplan–Meier survival plots versus time (in months) for patients stratified into high- (solid) and low- (dash) risk groups according to whether their estimated risk is above or below the median risk for the sample—risk values as shown in the left of Fig. 4. The stratification with LV-ROI mismatch is in red, and SAIF mismatch is in blue. (b) Comparison of risk values (red/blue for LV-ROI/SAIF) from logistic regression analysis of pathologic response. Patients who are found to respond to treatment are coded as “Yes” and nonresponders are coded “No.” The risk values are based on the formula in Table 2, as shown in the right of Fig. 4.

A direct comparison between the prognostic risk assessments corresponding to the survival and response models in Tables 1 and 2 is presented in Fig. 4. There is strong correlation between the risks evaluated using the LV-ROI and SAIF AIFs. As a further confirmation of the prognostic equivalence of the two AIFs, we compare Kaplan–Meier survival characteristics of patients stratified into high- and low-risk groups according to whether the model risk, calculated using coefficients in Table 1, is above or below the median risk for the sample. Note this stratification means that half the patients are designated as “high risk” and remaining patients are “low risk.” Their stratification produced using the risk calculation based on either LV-ROI or SAIF inputs are very similar (see Fig. 5). This is consistent with the quantitative analysis of likelihood and concordance reported in Table 1. A comparison between the risk distributions, calculated using coefficients in Table 2, among responders and nonresponders is presented in Fig. 5. The LV-ROI- and SAIF-based risks show similar separation between responders and nonresponders. Again, this is consistent with the quantitative analysis of model deviance reported in Table 2.

## 5 Discussion

This study is motivated by the increasing role of PET imaging in the clinical management of many cancer patients groups. Although most imaging with PET scanners is carried out in static mode, the widespread availability of the scanning technology now has opened up possibilities for more elaborate and potentially informative uses of its dynamic scanning capabilities. But there are a number of complexities associated with dynamic PET studies. At a technical level, more complete interpretation of the dynamic information requires a kinetic analysis using an appropriate compartmental model and the time course of activity in the arterial blood as an input function. In a clinical environment, image-based extraction of the arterial input function (AIF), as opposed to direct measurement by arterial or venous blood sampling, is usually a necessity. While several methods have been proposed for this,^4^^,^^6^^,^^7^^,^^21^ in the context of breast cancer studies, spillover from myocardial tissue uptake and nonarterially driven blood signals arising from the RV and the lungs complicate the extraction of the arterial time course.

The work presented explores the use of a statistical AIF extraction methodology based on a whole-body circulation model. The innovative element is that this model accounts for arterial, venous, and nonarterially driven signals—not just the arterially driven signal alone. Since nonarterial signals are particularly relevant in the context of breast cancer imaging, where the heart is the dominant blood pool in the field of view, it makes sense to evaluate the whole-body approach in this setting. Our group’s previous work has reported satisfactory performance for the extraction method in the context of cerebral imaging studies with $H_{2}O$ and FDG.^8^

The methodology used for blood extraction with $H_{2}O$ and FDG is calibrated using historical data from studies, in which directly sampled arterially curves for FDG and $H_{2}O$ were available. It is noteworthy that the nature of this process permits the combined use of data from studies with different injection protocols. For example, data from both 1- and 2-min infusions were used in our historical FDG studies. The whole-body circulation method allows these data to contribute to the overall statistical understanding of FDG whole-body circulation model parameters in past studies. In addition, if an infusion protocol was changed or even manual injection used, the extraction method would not need to be changed. This would not be the case if AIF extraction was based on a fixed template associated with a particular injection protocol.

The absolute scaling of the AIF is not resolved by the methodology. Importantly, however, the technique enables the AIF scaling to be achieved using either arterial or venous blood information. This flexibility is an advantage in settings where the complexities of direct arterial sampling would make venous measurement more appealing. In this work, a direct late-time LV-ROI value is used for scaling. No adjustment for myocardial spillover was used,^3^^,^^5^ such adjustment could improve the absolute quantitation accuracy. Whether this would impact the prognostic value of the derived blood flow-metabolism mismatch variable would need to be checked. Further studies involving direct (venous) blood sampling could shed light on the benefits of absolute quantitation.

Our results demonstrate that the extraction method satisfactorily recovers detailed kinetics and the blood flow-metabolism mismatch information from PET studies with $H_{2}O$ and FDG in breast cancer. This assessment is based on the prognostic utility of the derived blood flow-metabolism mismatch measure for early prediction of chemotherapy response and survival. There is no indication that the use of the automatic SAIF extraction method degrades the prognostic value of the studies. The ease of use of the SAIF extraction method and its reproducibility are its benefits. The current practice for our breast cancer blood flow-metabolism mismatch studies is based on a time course extracted from a carefully drawn ROI over a slowly moving part of the LV. This is often a technically challenging and time-consuming step. A reduced reliance on such expertise allows broader use of the blood flow-metabolism mismatch technique. This could also facilitate consideration of a multicentered imaging trial of the use of blood flow-metabolism mismatch studies in breast cancer patients.

## 6 Conclusion

SAIF is a reliable and reproducible AIF extraction method for diagnostic assessment of blood flow-metabolism mismatch from dynamic $H_{2}O$ and FDG PET studies in LABCa patients and can provide a helpful and robust tool for analysis of dynamic PET images studies where blood pool structures are included in the dynamic imaging field of view.
